# Feasibility of Using Low-Cost Motion Capture for Automated Screening of Shoulder Motion Limitation after Breast Cancer Surgery

**DOI:** 10.1371/journal.pone.0128809

**Published:** 2015-06-15

**Authors:** Valeriya Gritsenko, Eric Dailey, Nicholas Kyle, Matt Taylor, Sean Whittacre, Anne K. Swisher

**Affiliations:** Division of Physical Therapy, West Virginia University, Morgantown, West Virginia, United States of America; Universidad Europea de Madrid, SPAIN

## Abstract

**Objective:**

To determine if a low-cost, automated motion analysis system using Microsoft Kinect could accurately measure shoulder motion and detect motion impairments in women following breast cancer surgery.

**Design:**

Descriptive study of motion measured via 2 methods.

**Setting:**

Academic cancer center oncology clinic.

**Participants:**

20 women (mean age = 60 yrs) were assessed for active and passive shoulder motions during a routine post-operative clinic visit (mean = 18 days after surgery) following mastectomy (n = 4) or lumpectomy (n = 16) for breast cancer.

**Interventions:**

Participants performed 3 repetitions of active and passive shoulder motions on the side of the breast surgery. Arm motion was recorded using motion capture by Kinect for Windows sensor and on video. Goniometric values were determined from video recordings, while motion capture data were transformed to joint angles using 2 methods (body angle and projection angle).

**Main Outcome Measure:**

Correlation of motion capture with goniometry and detection of motion limitation.

**Results:**

Active shoulder motion measured with low-cost motion capture agreed well with goniometry (r = 0.70–0.80), while passive shoulder motion measurements did not correlate well. Using motion capture, it was possible to reliably identify participants whose range of shoulder motion was reduced by 40% or more.

**Conclusions:**

Low-cost, automated motion analysis may be acceptable to screen for moderate to severe motion impairments in active shoulder motion. Automatic detection of motion limitation may allow quick screening to be performed in an oncologist's office and trigger timely referrals for rehabilitation.

## Introduction

Breast cancer affects over 200,000 women in the US annually, and most of these women will undergo surgery as part of their treatment [1]. Shoulder motion impairments, including decreased range of motion (ROM), are common following surgery for breast cancer and can cause significant disability that can linger for years following treatment [2–4]. Symptoms that can limit shoulder motion, such as disuse, pain, numbness, and decreased strength, start to appear within days after cancer surgery and the percentage of patients that suffer from these symptoms tends to increase with time [4]. After the second week post-surgery more than 40% of patients showed a reduction in shoulder abduction of at least 10 degrees [5], and 37% of patients showed a reduction in shoulder flexion of at least 10 degrees [6]. Decreased shoulder ROM and strength can be a long-term problem reported up to 4 years following surgery [2,7,8]. Furthermore, radiation therapy has been implicated as a significant contributor to the reduction of ROM and strength of the shoulder joint in patients with breast cancer [9]. These ROM restrictions were also found to be significantly correlated with disability [10].

Physical therapy is an effective intervention for improving and overcoming arm morbidities after treatment for breast cancer. It has been shown that physical therapy intervention can improve ROM, improve function, increase strength, and decrease edema [2,7–9,11,12]. To identify patients who can benefit from physical therapy the current clinical gold standard is to employ a trained and skilled rehabilitation professional to measure shoulder ROM using goniometry. However, it is not feasible in many cancer centers to have a dedicated professional performing screening of all patients for motion impairments. Thus, development of an automated motion analysis system that could identify deficits in ROM would potentially enable impairment screening that could be performed during a routine clinic visit.

Current motion analysis systems, while very accurate, are expensive and require highly skilled personnel to operate and analyze data. The Kinect sensor, developed by Microsoft for video gaming, is a low-cost, small and portable system that can quickly record data on body position, which can be converted to joint angles. These data have been shown to be useful in assessing arm motor impairment in people with stroke [13], but their utility for assessing range of motion and detecting impairment after surgery has not been described. Therefore, the purpose of this study was to evaluate the feasibility of using the Kinect sensor to identify women with shoulder motion limitations following surgery for breast cancer.

## Methods

West Virginia University Institutional Review Board has approved the published research (protocol #1311129283). All participants provided written informed consent prior to any testing.

### Participant Recruitment

Participants were recruited from women diagnosed with breast cancer at the Mary Babb Randolph Cancer Center at West Virginia University. Eligible participants were women, age 18–80, with stage 0-III cancer and who undergone a unilateral mastectomy or lumpectomy and were not receiving rehabilitation services for their cancer. Participant recruitment was based on a convenience sample of 20 women returning for their follow up appointment one month after the surgery.

### Data Collection

Shoulder motion was recorded simultaneously using both a video camera and the Kinect sensor. Participants performed active shoulder motions while standing approximately 2 meters away from the recording equipment. Video camera was placed in front of the subject for abduction movements and on a side of the subject for flexion movements to minimize parallax distortion of joint angles. Video was captured with the standard 25 Hz frame rate. For half of all subjects, the Kinect sensor was placed at the same location as the video camera, i.e. in front of the subject for abduction movements and on the side of the subject for flexion movements. For the other half of all subjects, the Kinect sensor was left in front of the subject during both flexion and abduction movements. This was done to evaluate whether repositioning the Kinect sensor to observe movements at different angles would be necessary for better motion capture. Custom-written software played video clips illustrating each movement to be performed by the participant using the shoulder ipsilateral to the surgery. Participants performed three repetitions through the available, pain-free range of motion for: 1) active shoulder abduction (AA); 2) passive shoulder abduction (PA); 3) active shoulder flexion (AF); and 4) passive shoulder flexion (PF). During the passive movements, the participants were instructed to relax the ipsilateral arm, while using the contralateral arm to operate an over-door pulley to achieve shoulder flexion and abduction.

### Data Analysis

**Fig 1:**
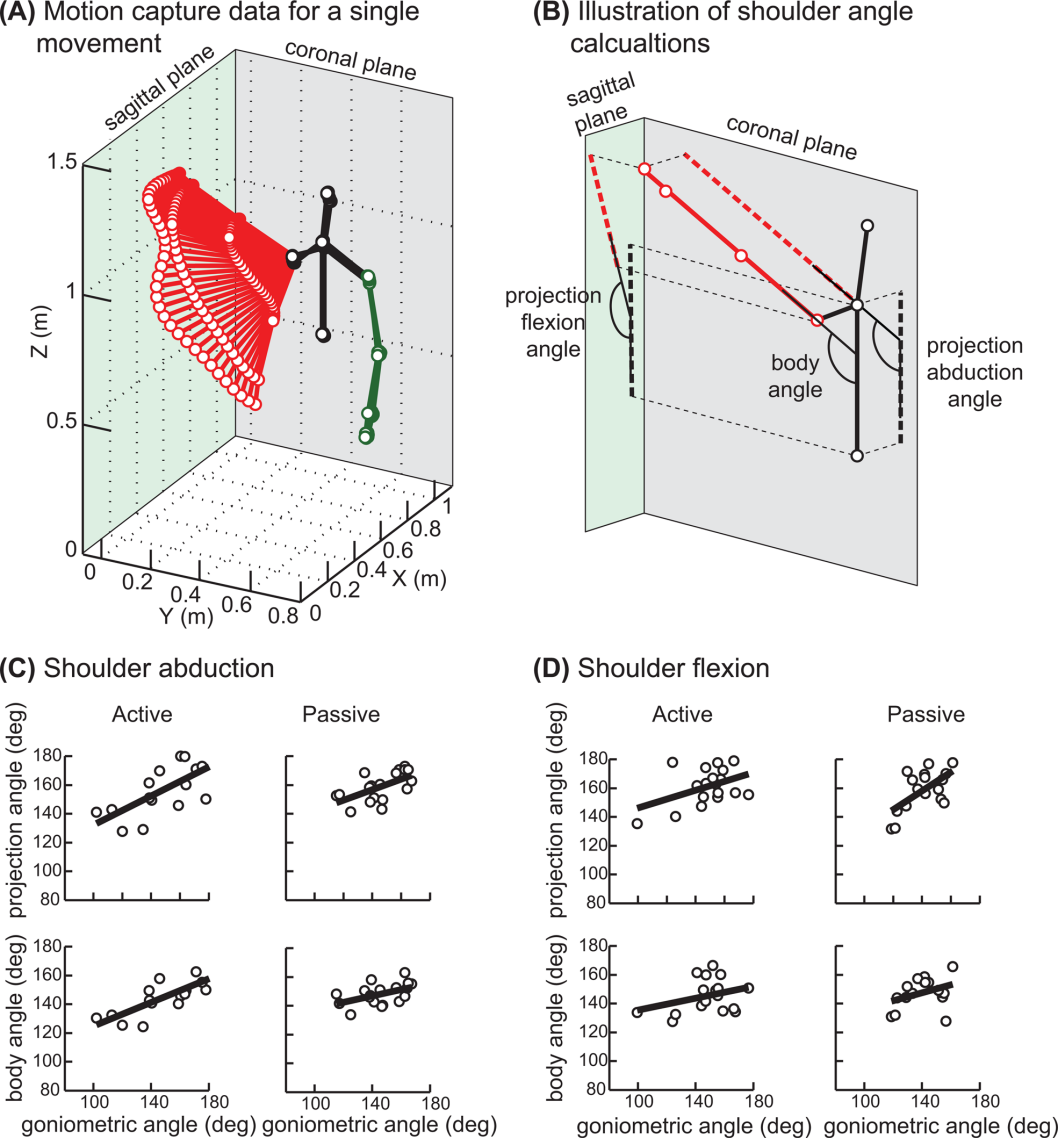
Motion illustration and associated shoulder angles. (A) Plot of the motion capture data from a single active abduction movement by a single subject. Lines connect body landmark coordinates on the upper body tracked by Kinect sensor in a single frame / moment in time, 30 frames per second were recorded. Black lines show trunk and head posture, red lines show right arm posture, and green lines show left arm posture. (B) Joint angle calculations illustrated on a single frame of motion capture data from the same movement as in A. Red line is a regression line through the right arm landmark coordinates; left arm landmark coordinates are removed for clarity. (C and D) Maximum joint angle measurements done using both methods described in the manuscript for abduction and flexion respectively. Circles show angles per subject averaged across 3 repetitions of the same movement; thick line is a regression. When fewer then 3 repetitions were recorded for a particular subject due to technical difficulties, the data was excluded from the analysis.

Goniometric measurements were made using a 6-inch goniometer placed on the appropriate landmarks displayed via projection of the video frame with the maximal shoulder excursion for each repetition of each motion [14]. Kinect motion capture data were processed in MATLAB (MathWorks, Inc.). The coordinates of multiple tracked skeletal landmarks were captured at 30 Hz (Fig 1A) and filtered using a second order Butterworth low-pass filter (cut-off at 6 Hz). Next, to reduce noise a straight line was fitted through the tracked points using least squares method. The fitted vector was used to calculate two types of maximal shoulder angles for each repetition of each movement. The first angle, termed "body angle", was between the trunk and the arm vectors (Fig 1B). The second angle, termed "projection angle", was based on projections of the arm vector onto the sagittal or coronal planes for flexion and abduction respectively. The projection angle measure was designed to match the apparent joint angle from video recording used for goniometric measurements. The maximal angle was averaged over 3 consecutive frames centered on the peak angle in order to compensated for the differences in frame rates between video and motion capture.

The relationship between angles from motion capture and goniometric measurements was analyzed by fitting linear regressions using least squares method. The regression coefficients for active movements were then used to identify participants with clinically significant shoulder ROM limitation of 30% or more (assuming normal motion of 180 degrees) using the following formula.

$$REF\;=\;(x_{AA}<180\times 0.7)\cap (x_{AF}<180\times 0.7)\cap (\left(x_{PA}<180\times 0.7\right)\cup \left(x_{PF}<180\times 0.7\right))$$

where ***REF*** is a Boolean referral vector with “true” values for participants with < 70% ROM; *x*_AA_, *x*_AF_, *x*_PA_, *x*_PF_ are ROM values for the corresponding movement types adjusted using regressions from Fig 1C and 1D. The false positive and false negative rates of detecting ROM limitations in a broader population was estimated using a statistically-equivalent artificial data of shoulder angles.

Data are reported as mean ± standard deviation values.

## Results

A total of 20 women, with a mean age of 60 ± 9 years participated in data collection. Most had undergone lumpectomy (n = 16) and were tested a mean of 18 ± 11 days following surgery. The Kinect system was easy and quick to use after brief training of the experimenter. Participants easily followed the video instructions shown during the test and performed movements with their affected arm in a consistent manner.

Maximal joint angles measured by each method are shown in Fig 1C and 1D. Overall, participants had good ROM in the shoulder ipsilateral to the breast surgery at the time of testing. Three participants (15% of all subjects) showed clinically-significant shoulder motion limitations and their goniometric measures were < 70% of normal ROM in active and passive shoulder abduction and flexion. As 50% of normal ROM indicates inability to lift the arm above the shoulder, the observed 30% or more ROM restriction would indicate moderate to severe shoulder impairment.

As expected, joint angles measured by the projection method were more closely matched to the goniometric measures from videos. This is supported by higher correlation coefficients between projection and goniometric angles vs. body and goniometric angles in all but one condition (Table 1, first and second columns). The power of all significant regressions across all 20 subjects was greater than 0.8. Overall, passive shoulder angles correlated less well with goniometry than active shoulder angles. This is likely due to the pulley system interfering with the motion capture algorithm of the Kinect sensor. Shoulder abduction angles were more closely correlated with goniometry than shoulder flexion angles in the active condition. However, when subjects with frontal positioning of the Kinect sensor were analyzed separately, the correlation between the projection and goniometric angle in the active flexion condition became significant (Table 1, column 3), although the power of this test was reduced to 0.4 due to smaller sample size. This shows that positioning the Kinect sensor to view subjects from the front results in the best quality of motion capture.

**Table 1:** Statistics of linear relationship between goniometry and joint angles from motion capture.

	Goniometry vs. projection angle All subjects		Goniometry vs. body angle All subjects		Goniometry vs. projection angle Last 10 subjects		Goniometry vs. body angle Last 10 subjects	
	r	p	r	p	r	p	r	p
**Active ROM abduction**	**0.698**	0.003	**0.794**	0.000	0.691	0.058	**0.786**	0.021
**Passive ROM abduction**	**0.599**	0.009	0.489	0.039	**0.706**	0.022	**0.768**	0.009
**Active ROM flexion**	0.438	0.069	0.323	0.190	**0.726**	0.017	0.580	0.079
**Passive ROM flexion**	**0.614**	0.009	0.356	0.127	0.651	0.030	0.660	0.027

Significant alpha with Bonferroni correction is 0.025. Significant Pearson product moment correlation coefficients (r) are in bold; p is probability of Type I error.

**Fig 2:**
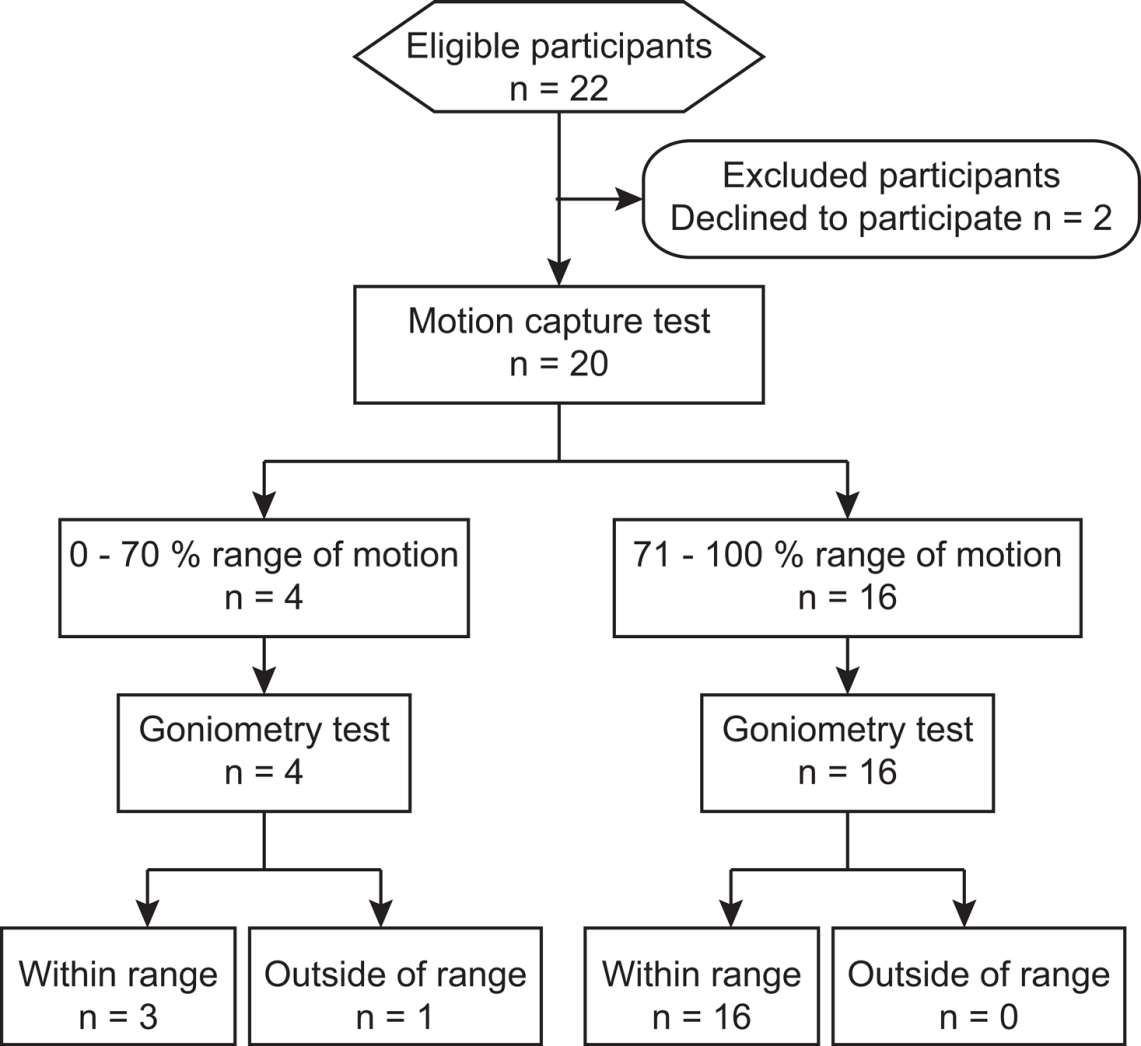
Flow diagram. The diagram shows the sequence of subject recruitment and testing with the associated numbers of participants (n) and detection accuracy.

Using the linear regressions and simple logical operations described in methods, the motion capture data enabled correct detection of visible shoulder motion limitations of all 3 subjects and incorrect detection of one subject that showed no visible shoulder motion limitations (Fig 2). To help address the feasibility of using this method to detect ROM limitations in a clinical population, we have estimated the false positive and false negative rates in a statistically-equivalent artificial data (see Methods). Detection of 30% ROM restriction observed in our experimental sample is associated with a false positive rate of 0.21 and a false negative rate of 0.02. Detection of more severe 40% ROM restriction is associated with a false positive rate of 0.08 and a false negative rate of 0.02. This finding suggests that it is feasible to use low-cost motion capture based on the Kinect sensor to detect reliably and automatically people with moderate to severe shoulder ROM restriction.

## Discussion

The purpose of this feasibility study was to determine if low-cost motion capture could be the basis of a simple automated method to detect shoulder motion impairment following breast cancer surgery. The strength of correlation with goniometry suggests that low-cost motion capture can provide measures of ROM that are comparable to those obtained by a skilled clinician. However, the maximal common variance between automated and clinical data was 60%, which indicates that motion capture by the Kinect sensor is noisy, a fact observed in other studies [13,15,16]. These studies have reported errors of similar scale for motion capture of whole body postures, isolated arm motion, and locomotion. Despite high variability of the motion capture data, our study results have shown that a simple combination of several angular measures enables reliable detection of moderate to severe shoulder motion restriction. This supports the feasibility of using this approach as part of an automated screening tool during routine follow-up clinical visits to identify people who have shoulder motion impairment and flag them for potential referral for physical therapy. Such a tool may also address the issue of limited access to care, as evidenced by a recent study reporting that only 33% of breast cancer survivors experiencing upper extremity problems receive physical therapy [17].

Another benefit of automated assessment tools is the objective data they provide that can be used to not only follow patient recovery or morbidity, but also evaluate treatment outcomes and help develop new treatments. Both appear to be lacking in the treatment of motion deficits in people with cancer [18]. Multiple studies have tested the usefulness of the Kinect sensor as a motion capture tool for clinical use with elderly people, stroke survivors, and people with Parkinson’s disease, cerebral palsy, and multiple sclerosis (for reviews see [19–22]). Several studies have shown that the motion capture data from the Kinect sensor can be used to obtain objective assessment of movement deficits [15–16,23–25]. Other studies have developed algorithms to use motion capture data from the Kinect sensor to detect falls in elderly [19,26]. The Kinect sensor has also been used to monitor objectively progress during traditional interventions, such as constraint induced therapy of stroke survivors [19,23–24]. There have also been novel virtual realty-based upper extremity rehabilitation programs developed with the Kinect sensor for people with cerebral palsy, stroke, and other motor disabilities [21–22,27–28]. However, the present study is the first to evaluate the feasibility of using low-cost motion capture for detection of shoulder impairment following recovery from breast cancer surgery. Results of our study suggest that the utility of low-cost motion capture for objective monitoring of post-surgical recovery of shoulder motion merits further study.

A sample of convenience was used, leading to the inclusion of women with different degrees of invasive surgery and different times between surgery and testing. We have observed that 15% of subjects showed clinically significant limitations in shoulder motion at the time of testing. Despite the heterogeneity of our sample, this is consistent with the time-course of arm morbidity reported in women with sentinel node-negative breast cancer [4].

Changing the location of the Kinect sensor during recordings of flexion movements increased the correlation between goniometric and projection angles, but the power of this relationship was low. However, the detection algorithm for identifying shoulder impairment was tested on all 20 subjects recruited for the study, regardless of the Kinect sensor placement. The algorithm performed well as evidenced by the low false positive and false negative rates. Therefore, our conclusion that it is feasible to use the low-cost motion capture for detection of moderate to severe shoulder ROM limitation is valid.

The method of obtaining goniometric joint angles described here relies on video recording of the subject motion, rather than the “gold-standard” measurement of actual joint angles while the subject holds her arm in the maximal ROM position. This was done to maximize the similarity between the goniometric and motion-capture-based angles by obtaining both measures from the same motion. Furthermore, only the subject needed to be visible to the Kinect sensor for best motion capture. The presence of the therapist in the Kinect sensor view during recording would reduce the quality of motion capture simply by interfering with the image recognition algorithm of the sensor, similarly to the way the pulley system did. While the video-based method of obtaining goniometric angles is untested and prone to parallax problems, we have taken steps to minimize foreseen limitations of this method as described in the methods. Our results show several high correlations between the goniometric and projection angles. Therefore, we are confident in the validity of using video for goniometry in this study.

## Conclusions

Using low-cost motion capture to measure ROM is a promising screening tool that can be deployed prior to professional medical assessment to detect shoulder motion impairments in women with breast cancer and to identify those who would most likely benefit from rehabilitation to remediate those impairments.
